# Contribution of the Surface Treatment of Nanofibrillated Cellulose on the Properties of Bio-Based Epoxy Nanocomposites Intended for Flexible Electronics

**DOI:** 10.3390/ijms24076544

**Published:** 2023-03-31

**Authors:** Adriana Nicoleta Frone, Cătălina Diana Uşurelu, Gabriela Mădălina Oprică, Denis Mihaela Panaitescu, Augusta Raluca Gabor, Cristian-Andi Nicolae, Florin Ciuprina, Celina Maria Damian, Florentina Monica Raduly

**Affiliations:** 1 Polymer Department, National Institute for Research and Development in Chemistry and Petrochemistry, 202 Splaiul Independentei, 060021 Bucharest, Romania; catalina.usurelu@icechim.ro (C.D.U.); madalina.oprica@icechim.ro (G.M.O.); panaitescu@icechim.ro (D.M.P.); raluca.gabor@icechim.ro (A.R.G.); cristian.nicolae@icechim.ro (C.-A.N.);; 2 ELMAT Laboratory, Faculty of Electrical Engineering, University Politehnica of Bucharest, 313 Splaiul Independentei, 060042 Bucharest, Romania; florin@elmat.pub.ro; 3 Advanced Polymer Materials Group, University Politehnica of Bucharest, 1-7 Gh. Polizu Street, 011061 Bucharest, Romania; celina.damian@upb.ro

**Keywords:** nanofibrillated cellulose, surface treatment, epoxidized linseed oil, bio-based epoxy nanocomposites, flexible electronics

## Abstract

The growing interest in materials derived from biomass has generated a multitude of solutions for the development of new sustainable materials with low environmental impact. We report here, for the first time, a strategy to obtain bio-based nanocomposites from epoxidized linseed oil (ELO), itaconic acid (IA), and surface-treated nanofibrillated cellulose (NC). The effect of nanofibrillated cellulose functionalized with silane (NC/S) and then grafted with methacrylic acid (NC/SM) on the properties of the resulted bio-based epoxy systems was thoroughly investigated. The differential scanning calorimetry (DSC) results showed that the addition of NCs did not influence the curing process and had a slight impact on the maximum peak temperature. Moreover, the NCs improved the onset degradation temperature of the epoxy-based nanocomposites by more than 30 °C, regardless of their treatment. The most important effect on the mechanical properties of bio-based epoxy nanocomposites, i.e., an increase in the storage modulus by more than 60% at room temperature was observed in the case of NC/SM addition. Therefore, NC’s treatment with silane and methacrylic acid improved the epoxy–nanofiber interface and led to a very good dispersion of the NC/SM in the epoxy network, as observed by the SEM investigation. The dielectric results proved the suitability of the obtained bio-based epoxy/NCs materials as substitutes for petroleum-based thermosets in the fabrication of flexible electronic devices.

## 1. Introduction

Epoxy resins are one of the most widely used thermosetting polymers, which become irreversibly hardened when cured by heating and/or by the addition of crosslinking agents [1,2]. Today, epoxy resins account for approximately 70% of the thermosets market, excluding the polyurethane thermosets [3]. Endowed with high tensile and compression strengths, excellent adhesiveness to a variety of substrates, good electrical insulation properties, high corrosion and chemical resistances, and superior thermal stability, epoxy resins have found multiple uses in the coatings and adhesives industries, electronics, civil engineering, and as matrices for high-performance composites [4]. Unfortunately, nowadays, 90% of the epoxy resins produced around the world are still based on diglycidyl ether of bisphenol A (DGEBA), which results from the polycondensation of bisphenol A (BPA) and epichlorohydrin (ECH), two fossil-based precursors [5]. BPA and ECH are potential mutagens and carcinogens [6,7,8] and, owing to its structural similarity to estrogen, BPA is believed to be an endocrine disruptor with adverse effects on the growth, regeneration, embryonic development, level of energy, and fertility of living beings [9]. In addition to their non-renewable origin and toxicological issues, a main drawback of the DGEBA epoxies is their brittleness after curing [10,11]. In the last two decades, a myriad of compounds that originate from renewable resources, including vanillin, eugenol, cardanol, resveratrol, guaiacol, tannic acid, gallic acid, and vegetable oils, have been studied as precursors for obtaining bio-based epoxies [2,12,13,14,15,16]. From these, vegetable oils received increased attention due to their easy availability, low price, biodegradability, non-toxicity, [13], and easy conversion to their epoxidized derivatives thanks to the reactive C=C double bonds from their structure [2]. In addition, the long flexible alkyl chains of vegetable oils open the possibility of obtaining epoxies with improved flexibility and toughness [14].

To achieve solid, dimensionally, and thermally stable three-dimensional networks, epoxy resins need to be cured in the presence of crosslinking agents, either at room temperature or at elevated temperatures [17]. However, common crosslinking agents for epoxy resins are aliphatic and aromatic amines or anhydrides, which are toxic and environmentally unfriendly [7,18]. Several alternatives to standard crosslinking agents have been proposed to date [16,19,20]. Citric acid (CA), a naturally occurring compound, holds in its structure three carboxyl groups capable of participating in esterification reactions with the hydroxyl groups of the polymers [21]. Sahoo et al. studied the curing of bio-based epoxy resins deriving from castor and linseed oils with CA [19]. The elongation at break of the two CA-cured epoxy resins obtained from vegetable oils was much better than that of the CA-cured DGEBA epoxy at the expense of the tensile strength and modulus. Due to its higher epoxy value, the thermoset based on epoxidized linseed oil showed better thermal stability and storage modulus than the one based on epoxidized castor oil [19]. Tannic acid (TA), a polyphenol with an aromatic ring structure, is a suitable candidate to form a stable network with the epoxy resin. TA is recommended for its biobased nature, lack of toxicity and low cost, and has the advantage that it is already industrially isolated. Reinhardt et al. obtained a fully bio-based epoxy by milling TA into a fine powder and incorporating the powder in ELO, without the need for a solvent, followed by curing. The obtained epoxy material was characterized by good mechanical and thermal properties, similar to that of high-performance petrochemical-based epoxy resins [16]. However, the high flexural strength and modulus were achieved at the expense of flexibility.

A good alternative to standard crosslinking agents comes in the form of itaconic acid (IA), a dicarboxylic acid that can form crosslinked structures with the epoxy groups through its carboxyl groups. IA is a fully sustainable compound and one of the top building blocks of the chemical industry. Its bio-based synthesis is simple, consisting of carbohydrate fermentation in the presence of fungi from the *Aspergillus* family [22]. Another advantage of IA is the presence of a vinyl group, a potential site for polymerization. In particular, Naderi et al. used IA for synthetizing an internal crosslinking agent with glycidyl methacrylate and obtained superabsorbent hydrogels with improved strength [23].

From all epoxidized vegetable oils, epoxidized linseed oil (ELO) is particularly attractive as a precursor for producing bio-based epoxy with increased flexibility and toughness due to its superior reactivity and higher number of epoxy groups in its structure [16] as compared to other epoxidized vegetable oils, such as the epoxidized soybean oil (ESO) and cottonseed oil. Di Mauro et al. synthesized bio-based epoxy resins starting from ESO and ELO, using itaconic acid as crosslinking agents in the absence of any initiator [20]. For comparison, they also obtained formulations based on a synthetic epoxy resin precursor, i.e., DGEBA and IA as a crosslinker. The formulation based on DGEBA and IA showed higher reactivity in the curing reaction as compared to the formulations based on ESO and IA or ELO and IA. Nevertheless, from the two epoxidized vegetable oils-based systems, ELO/IA displayed better reactivity in the crosslinking reaction due to the higher number of epoxy groups from the ELO’s structure. The dynamic mechanical results (DMA) revealed that the thermosets based on ELO/IA and ESO/IA possessed higher flexibility as compared to the system based on DGEBA/IA, exhibiting much lower T_g_ values of 37 °C and 13 °C as compared to the 63 °C recorded for the DGEBA/IA thermoset. The higher T_g_ value obtained for the DGEBA/IA system was attributed to the rigid aromatic rings from the DGEBA’s structure and its higher crosslinking density [20].

The room for improvement in the properties of epoxy resins can be filled with nanocellulose fibers. Cellulose, a polysaccharide with straight chains of D-glucose connected by β-1,4-glycosidic linkages, represents the most abundant terrestrial biopolymers in the world and is a source of nanocellulose [24,25]. Due to its versatility regarding the geometry, from (nano)fibers to (nano)particles, the multitude of isolation sources, and superior properties in terms of renewability, biocompatibility, biodegradability and lowcost, cellulose has been an attractive material choice for developing new bio-based products for a variety of applications [26,27]. As a consequence of its nanodimensions, nanocellulose (NC) possesses a high surface area, which may allow for the formation of stronger interactions with the polymer matrix. The valuable properties of nanocellulose, such as its all-around availability, renewability, biodegradability, and good mechanical properties, come to strengthen those of the epoxy resins. NC has been intensively studied as a reinforcing agent in bio-based polymers such as poly(lactic acid) [28,29], polyhydroxyalkanoates [30,31], poly(butylene succinate) [32], and starch [33,34], leading to fully-biobased materials with improved mechanical properties and, in some cases, with better thermal properties. However, a smaller number of studies were dedicated to the reinforcement of common epoxy resins with cellulose nanocrystals or nanofibers [17,35,36,37,38]. One problem in the case of nanocomposites based on hydrophobic polymer matrices and hydrophilic fillers is the weak interaction at the interface that leads to the poor dispersion of the nanofiller [37]. This drawback can be corrected via the chemical modification of NC using the multitude of reactive hydroxyl groups from its surface [37,39]. In particular, cellulose nanofibers extracted from filter-paper were grafted with polyethyleneimine (PEI) to improve the interfacial adhesion between NC and the bisphenol A type epoxy resin [37]. The increased interfacial crosslinking density in these nanocomposites was reflected by the strong increase in tensile strength and modulus. A similar effect was observed for a bisphenol A type epoxy resin reinforced with cellulose nanocrystals (CNCs) grafted with lauric acid [39]. Good mechanical properties were obtained for the nanocomposites with slightly modified CNCs (degrees of substitution of 0.2 and 0.8). These nanocomposites also exhibited a slight change in flexibility [39]. In another attempt, microfibrillated cellulose was functionalized with triethoxy(3-glycidyloxypropyl)silane (GPS) and used as a reinforcing agent in a bisphenol A type epoxy resin [40]. A strong increase in the impact strength was obtained for the epoxy resin containing GPS-treated NC as fillers as compared to the one recorded for the epoxy matrix containing untreated NC. GPS-treated NC also led to epoxy nanocomposites, showing better tensile strength and modulus [40]. Triblock copolymers surfactants containing poly(ethylene oxide) and poly(propylene oxide) blocks were also assessed for strengthening the NC–bisphenol A epoxy interface [41].

Only a few works deal with bio-based epoxy resins reinforced with NC or cellulose fibers [42,43,44]. Shibata et al. prepared bio-based epoxy resins from ESO by using TA as a curing agent [42]. The composites obtained from these bio-based epoxy systems and 5–11 wt% cellulose fibers (CF) with submicron width showed improved mechanical properties; an increase in tensile strength by 50% and a 3-fold increase in modulus along with a drastic reduction in the elongation at break were observed for the composite with 11 wt% CF [42]. Weaker improvements in the mechanical properties were reported for bio-based epoxy composites containing similar concentration of CF but based on sorbitol glycidyl ether and TA [43]. A terpene–maleic ester type epoxy resin reinforced with 0.5–8 wt% cellulose nanowhiskers (CNW) showed improved mechanical properties [44]. The addition of only 1 wt% CNW led to more than 2-fold increase in the Young’s modulus and a 50% increase in the tensile strength of the terpene–maleic ester epoxy resin. However, the elongation at break of the same composite suffered a 12-fold reduction compared to the unmodified epoxy [44], which characterizes a very stiff material. Therefore, in this study, an epoxidized vegetable oil and a bio-based crosslinker were used to obtain the epoxy resin. Epoxy resins were obtained from ELO and IA using small amounts of grafted and ungrafted NCs for balancing the stiffness and flexibility of these systems. For improving the interface properties, NC was surface-modified firstly by treatment with a silane and then by grafting polymerization with methacrylic acid. To the best of our knowledge, bio-based epoxy nanocomposites consisting of ELO resin, IA as a cross-linking agent, and NCs as filler have not yet been reported. The structural, morphological, thermo-mechanical, and electrical properties of the resulting nanocomposites were determined for assessing the performances of the new fully-bio-based nanomaterials. The epoxy nanocomposites with balanced mechanical properties obtained in this study are designed for engineering applications, and especially for flexible electronic devices.

## 2. Results and Discussion

**Figure 1:**
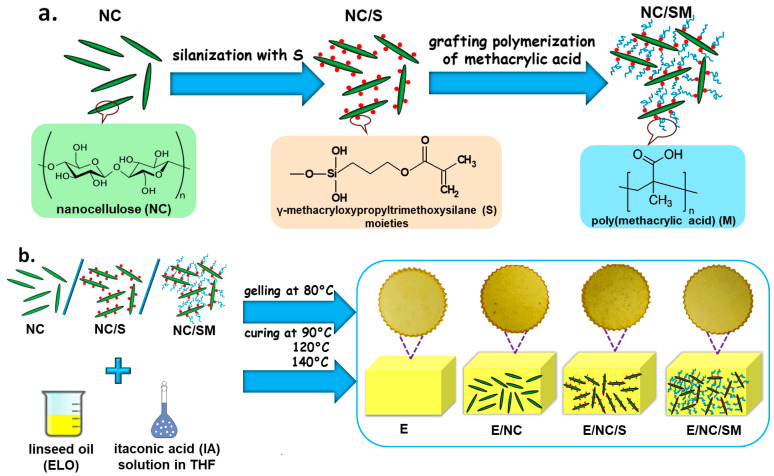
Graphical representation of NC grafting (**a**) and of the obtaining procedure for the ELO/NC nanocomposites (**b**).

The schematic representations of the surface modification of NC and the preparation route of the cured epoxy nanocomposites are shown in Figure 1a,b.

### 2.1. FT-IR Spectra of Pristine Materials and Epoxy/NC Nanocomposites

**Figure 2:**
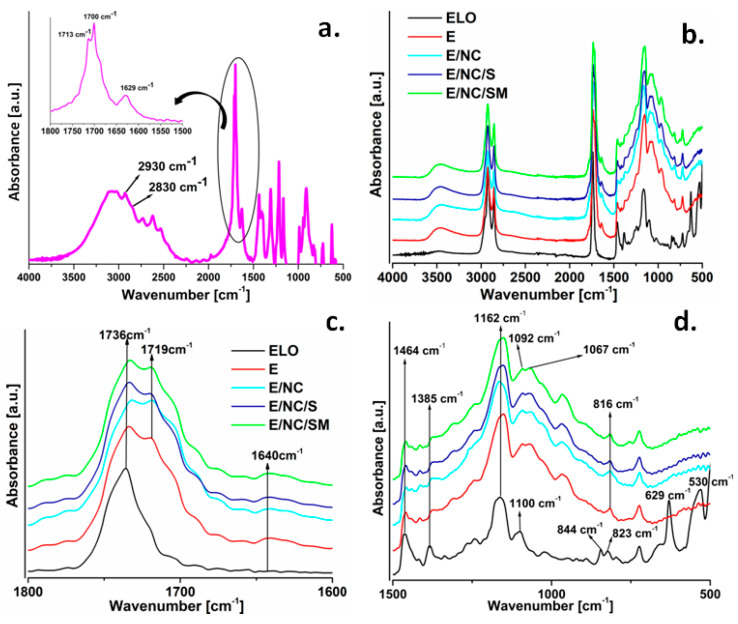
ATR-IR spectra of neat IA (**a**), neat ELO, cured neat epoxy matrix, and epoxy nanocomposites at wavenumber ranges between 4000 cm^−1^ and 500 cm^−1^ (**b**), 1800 cm^−1^ and 1600 cm^−1^ (**c**), and 1500 cm^−1^ and 500 cm^−1^ (**d**).

FT-IR was used to investigate the changes induced by the addition of differently treated NCs on the structural properties of cured epoxy nanocomposites (Figure 2). The FT-IR spectra of pristine materials, itaconic acid (IA), and epoxidized linseed oil (ELO) are shown in Figure 2a,b. The broad absorption band in the range of 3300–2300 cm^−1^ from the spectrum of IA (Figure 2) comprises bands assigned to the O-H stretching vibrations (3000–3100 cm^−1^) and -C-H asymmetric (2930 cm^−1^) and symmetric (2830 cm^−1^) stretching bands of CH_2_ groups [20,45]. The bands at 1700 and 1713 cm^−1^ are related to the C=O stretching, and that at 1630 cm^−1^ to the C=C stretching vibrations from IA’s chemical structure [46].

The spectrum of neat ELO shows absorption bands at around 3500 cm^−1^, which correspond to the stretching vibrations of the O-H groups, a band assigned to the carbonyl (C=O) stretching from the ester functionality (1736 cm^−1^), bands associated with the -CH asymmetric and symmetric bending vibrations from the CH_2_ and CH_3_ groups (1464 and 1385 cm^−1^), and the oxirane C-O twin bands at 823 cm^−1^ and 844 cm^−1^ [20,47] (Figure 2).

According to literature, the reactions between the epoxy groups from resins and the carboxyl groups of dicarboxylic acids are quite complex, and esterification, etherification, condensation, and hydrolysis are all possible [48]. Thus, several changes were detected in the close-up spectra of the thermally cured neat epoxy system (E) and epoxy nanocomposites (Figure 2c,d).

The bands assigned to the stretching vibrations of OH groups from 3500 cm^−1^ increased in intensity in the cured samples, indicating that the carboxyl groups of itaconic acid determined the opening of the epoxy cycles with the formation of new OH groups [49]. For comparing these signals, the intensity of the band was normalized, using the C-H stretching vibrations at 2830–2900 cm^−1^ as an internal reference. A new band at 1719 cm^−1^ appeared after curing, which could be assigned to a newly formed ester C=O bond (Figure 2c) [20,22].

Moreover, the disappearance of the bands attributed to the specific signals of oxirane groups (823, 844, and 629 cm^−1^) from the cured epoxy matrix and epoxy nanocomposites’ spectra are a clear demonstration of the formation of ELO-based 3D networks following the crosslinking with IA (Figure 2d). Interestingly, the band at 1100 cm^−1^ assigned to the C-O stretching vibrations of O=C-O from ELO splits into two new bands (1092, 1067 cm^−1^) after the curing reaction as a consequence of the formation of new types of covalent C-O-C bonds. The newly emerged bands are typical of the ether C-O-C bands formed in accordance with the epoxy–acid curing mechanism [47]. These findings are valid for the neat epoxy system and the epoxy/NCs nanocomposites, regardless of the NCs type.

All the above observations are solid proof of the efficiency of the bio-derived IA as a crosslinker for the ELO matrix and its nanocomposites with NCs and confirm the occurrence of the curing process.

### 2.2. TMDSC Analysis of the Obtained Epoxy/NC Nanocomposites

**Figure 3:**
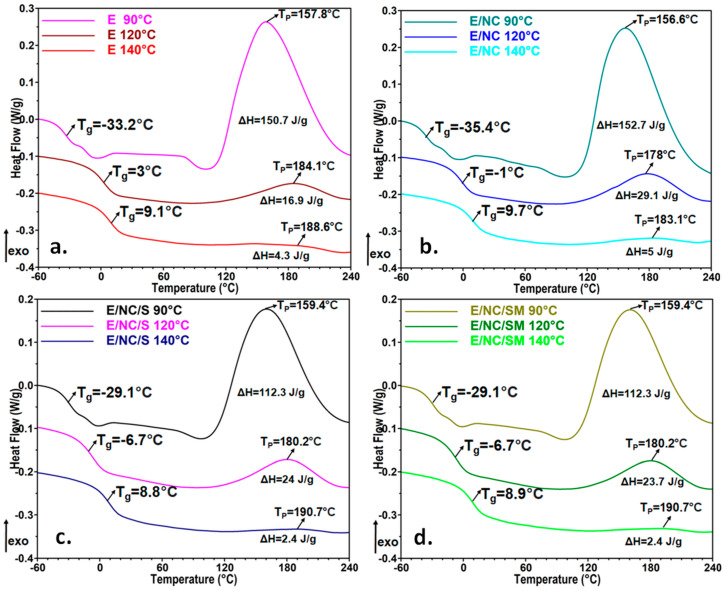
DSC thermograms of the curing process of the neat epoxy system (**a**) and nanocomposites containing ungrafted (**b**) and grafted NCs (**c**,**d**).

The suitability of IA as a crosslinking agent, together with the influence of nanocellulose addition upon the curing behavior of the ELO bio-resin, was assessed by MDSC. Figure 3a–d show the thermal behavior of the epoxy resins after each step of the curing protocol and the main parameters i.e., the glass transition temperature (*T_g_*), the maximum peak temperature (*T_P_*), and corresponding enthalpies (Δ*H*).

All the DSC curves displayed a single exothermic peak corresponding to the ring-opening reaction between the epoxy functions of ELO and the carboxyl groups of IA (Figure 3a–d). The *T_P_* of the DSC exothermic curves can provide important information regarding the efficiency of the bio-derived IA as a curing agent, being oftentimes taken as an indicator for the reactivity of the constituents in a curing reaction, a lower peak corresponding to a higher reactivity of the components [22]. Moreover, both the neat epoxy matrix and its nanocomposites containing NCs displayed a single *T_g_*, and the *T_g_* values decreased with increasing the curing temperature (Figure 3a–d).

From the DSC curves of all studied samples, one can observe that the applied thermal curing protocol was effective, leading first to the decrease and then to the almost complete disappearance of the *T_P_* peak (Figure 3a–d). This behavior denotes the consumption of the reactive groups involved in the crosslinking mechanism and is strong evidence of a complete curing reaction. *T_P_* is clearly dependent on the increase in both curing temperature and time and it is accompanied by a dramatic decrease in the corresponding enthalpies values (Figure 3a–d). The values of the *T_P_* belonging to the neat epoxy matrix are comparable with already reported literature data concerning the crosslinking of different vegetable oils with C5 and C6 dicarboxylic acids [20,48]. *T_P_* values in the range 155–158 °C were reported by Di Mauro et al. who studied the reactivity of linseed and soybean epoxidized vegetable oils in the presence of IA. In this case, ethanol was employed as a solvent for the ELO resin and the cure protocol involved higher temperatures (150 °C and 180 °C) [20]. A *T_P_* value of ~178 °C, closer to those of the samples in this study was found by Ding et al. for the ELO/adipic acid epoxy system in the presence of 4-*N,N*-dimethylaminopyridine (DMAP) as an accelerator [48].

Further, the addition of NCs did not significantly affect the curing process, which proceeded similarly to that of the neat epoxy matrix but influenced the exothermic peak. Therefore, a slight decrease in the *T_P_* with about 6 °C was noticed in the case of the epoxy nanocomposite containing unmodified NC against the neat epoxy matrix, especially in the last step of the curing reaction, which suggests a higher reactivity of the E/NC nanocomposite. This may be due to the hydroxyl groups from the NC’s surface, which could accelerate the curing reaction between the epoxy groups and IA through the opening of the oxirane cycle and the generation of new -OH groups that further interact with the IA structure [22]. This assumption is also supported by the higher value calculated for the curing enthalpy of the E/NC samples, because the formation of a higher number of bonds (even H bonds) will lead to an increased exothermal effect. Opposite behavior was observed for the nanocomposites containing grafted NCs (Figure 3c,d). In this case, the number of free hydroxyl groups was reduced as a result of NCs chemical grafting, and the above-mentioned mechanism was hindered. Indeed, the addition of chemically modified NCs in the epoxy matrix results in a small increase (with less than 2 °C) of the *T_P_* value during the curing process (first and third stage of curing) as against neat sample. Similarly to the *T_P_* variation, a slight reduction in the *T_g_* values was found for the epoxy nanocomposites containing grafted NC [50]. Thompson et al. attributed the reduction in the *T_g_* of an epoxy composite containing 1 wt% cellulose nanocrystals modified with methacryloyl chloride (M/CNCs) to the fact that M/CNCs can act as a physical barrier within the epoxy matrix, reducing the curing efficiency of the crosslinking system or to the improved dispersion of the M/CNCs inside the matrix, which could have further hindered the curing process [51].

### 2.3. TGA Analysis of Neat Epoxy System and Epoxy/NC Nanocomposites

**Figure 4:**
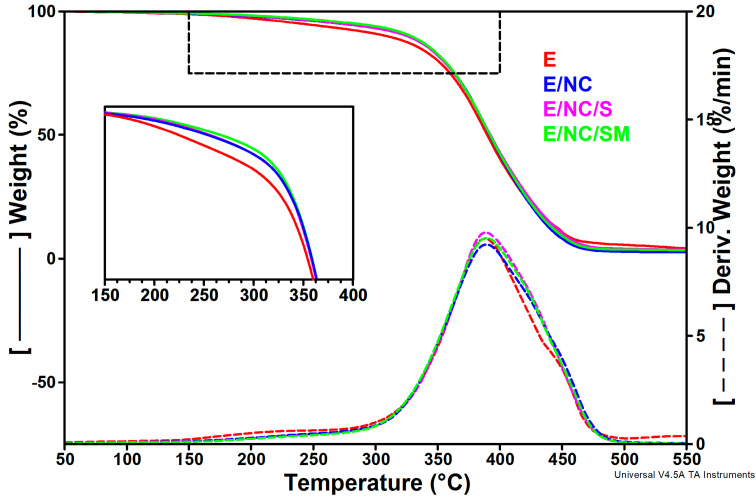
TGA, DTG and inset from 150 to 400 °C for the neat epoxy system and epoxy nanocomposites.

The thermal stability of the neat epoxy matrix and its nanocomposites containing NCs was assessed by TGA analysis and depicted in Figure 4, where the integrated (TGA) and derivative (DTG) forms are shown. The related thermal parameters i.e., the temperatures at 5% weight loss (T_d,5%_) and at the maximum degradation rate (T_d,max_), the weight loss at temperature of 200 °C (WL_200°C_), and the residue at 750 °C (R_750_) are summarized in Table 1.

The neat epoxy system and its nanocomposites containing NCs present similar degradation behavior, with two degradation processes being noticed in the thermal degradation profiles. No separate degradation stage of NCs was observed in the TGA or DTG thermograms of the epoxy nanocomposites, regardless of the NCs type. The first degradation stage from 150 °C to 300 °C is probably due to the labile C-O type bonds from the molecular structures with higher mobility not being embedded within the epoxy network [47,48,52]. The main degradation step, which is placed between 330 °C and 480 °C, results from the chain cleavage of the ester groups in the ELO epoxy resin structure [53,54]. The breakage of the ester groups formed through the ring-opening reactions between the epoxy functional groups and the carboxyl ones occurred above 480 °C, and this is more evident in the matrix without NCs (Figure 4) [47,48].

**Scheme 1:**
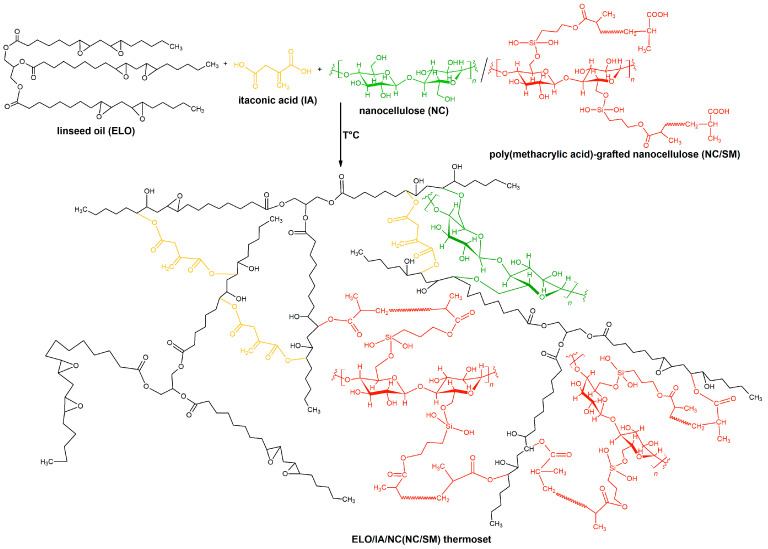
Illustration of proposed crosslinking mechanism of ELO resin with IA in the presence of grafted (NC/SM) and ungrafted (NC) nanofibrillated cellulose.

The presence of NC and its treatment influenced the thermal stability of the nanocomposites, especially at temperatures below 300 °C. All nanocomposites, regardless of the NCs type, exhibited an important improvement in the thermal stability, T_d,5%,_ increasing with up to ~44 °C as compared with the neat epoxy matrix (Figure 4, Table 1). Additionally, small mass loss values at 200 °C were obtained for the nanocomposites as against the epoxy matrix without NCs (Table 1). In the case of ungrafted NC, the enhancement of the thermal stability can be explained by the active hydroxyl groups on the surface of NC that can be involved in the crosslinking reactions along with the carboxyl groups of IA, increasing the crosslinking efficiency. For the NC/SM, the poly(methacrylic acid) grafts present on its surface could react with the epoxy system during the cross-linking process, with the formation of a more complicated and stable network (Scheme 1) [51]. In both cases, a strong interfacial adhesion was formed between the NCs and the epoxy network [51]. Therefore, this high thermal stability of nanocomposites can be attributed to effective interactions between NCs and the epoxy matrix [55].

Improved thermal properties were also reported by Pandurangan et al., when cellulose nanofibers derived from banana fibers were incorporated in low amounts (<3 wt%) into a commercially petroleum-based epoxy resin [56]. The authors explained the positive results through the nanolevel dispersion of the CNF particles inside the epoxy polymer matrix.

On the other hand, the T_d,max_ values of the nanocomposites were quite close to the one observed for the neat epoxy matrix. Therefore, regardless of the surface treatment, the NC did not influence the T_d,max_, i.e., the temperature corresponding to the decomposition of the polymer with the highest rate. With regards to the residual content, a very small amount of char residue was obtained for the neat epoxy, and slightly higher amounts were detected for the nanocomposites due to the presence of NCs and the additional reactions discussed above.

The above-discussed results showed that the addition of NCs improved the thermal stability of the epoxy-based materials, thus increasing the chances of using vegetable oil-based epoxy systems in fields that require resistance to high temperatures. From Table 1 it is evident that, among all samples, the E/NC/SM nanocomposite exhibited the highest thermal stability.

### 2.4. DMA Analysis of Neat Epoxy System and Epoxy/NC Nanocomposites

**Figure 5:**
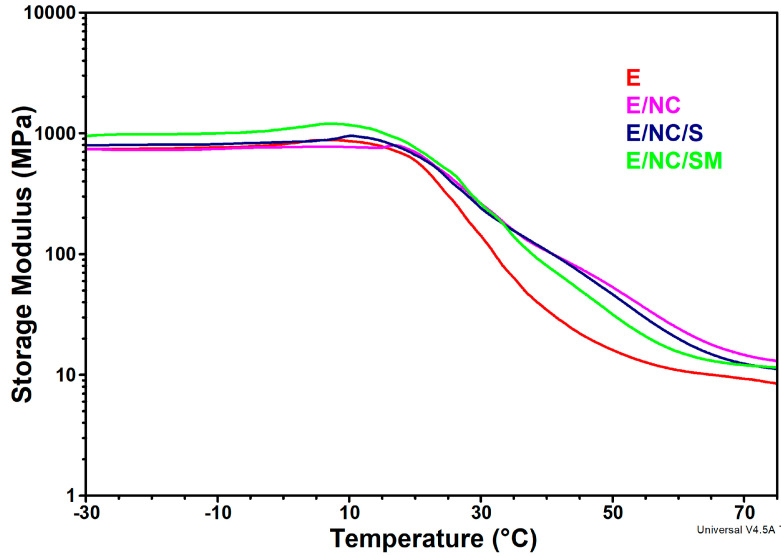
DMA storage modulus (E’) for the neat epoxy system and epoxy nanocomposites.

The E’ vs temperature curves of all materials are resembling those of amorphous thermoset polymers, presenting three stages: a glassy plateau, an *α* transition region (or “glass transition region”) where the E’ drops significantly as a consequence of the increase in the chain segments mobility near the glass transition temperature, and a rubbery plateau (Figure 5).

As indicated in Table 2, in the glassy domain (*E*’_−25°C_ and *E*’_0°C_), the lowest E’ was recorded for the E/NC nanocomposite. The reduction in the E’ modulus for the E/NC nanocomposite as compared to the neat epoxy may be attributed to an insufficient interfacial adhesion between the hydrophilic NC and the hydrophobic epoxy matrix, which did not allow for an efficient load transfer from the matrix to the reinforcing agent.

On the contrary, the incorporation of surface-modified CNs had a strong reinforcing effect in both the glassy and the glass transition regions (Table 2). Thus, an improvement in the E’ of 7.4% and 31%, respectively, was recorded at −25 °C for the E/NC/S and E/NC/SM nanocomposites as compared to the neat epoxy, showing that the surface-modified NCs increased the epoxy matrix’s ability to undergo mechanical stress with recoverable viscoelastic deformation [57].

The increase in the E’ following the addition of modified NCs may be an expression of the good compatibility between the surface-modified NCs and the epoxy matrix due to the favorable interactions established between the polymethacrylate grafts of the modified NC and the epoxy matrix, as previously demonstrated by the thermal analysis results. Thus, the good adhesion at the interface between the nanofiller and the epoxy matrix facilitated a good load transfer from the epoxy matrix to the rigid NCs and led to nanocomposites with enhanced homogeneity and superior stiffness.

**Figure 6:**
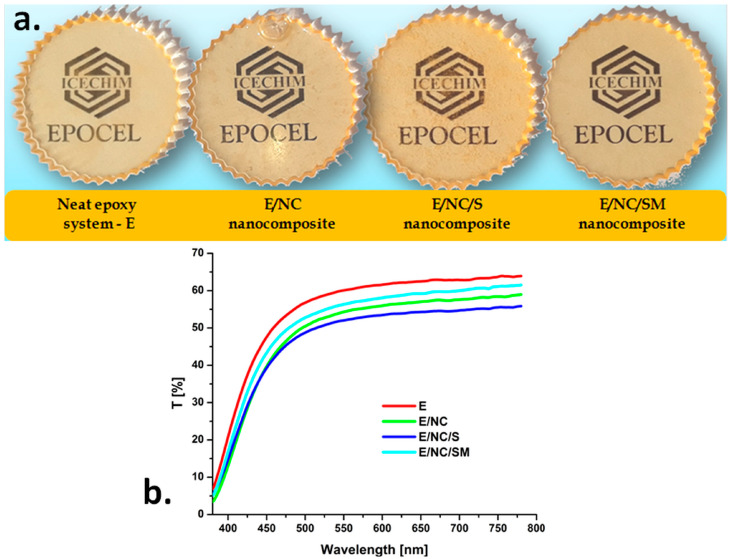
Images of the cured neat epoxy matrix and epoxy/NC nanocomposites indicating their transparency (**a**); Light transmittance measurements versus the wavelength for the cured neat epoxy matrix and epoxy/NC nanocomposites (**b**).

At room temperature, an increase in the *E*’_25°C_ of around 44%, 30%, and 60% was obtained for the E/NC, E/NC/S, and E/NC/SM nanocomposites over the neat epoxy matrix, while at higher temperatures (i.e., at 50 °C) the increase was even more significant, being 232%, 190%, and 98%. In both cases, the higher E’ values can be ascribed to the NCs which act as physical hindrances, restricting the free movement of the epoxy matrix chain segments. Again, the nanocomposite containing NC/SM stands out as compared with the other nanocomposites due to its higher stiffness. In this case, we might assume that NC/SM has an improved distribution in the epoxy matrix which leads to an improved toughening mechanism [58]. For verifying this assumption, light transmittance measurements were performed, and the results confirmed the better dispersion of NC/SM nanofibers in the epoxy matrix as compared with the nanocomposite containing unmodified or silane modified NC (Figure 6b). The light transmittance of polymer composites depends on many factors, such as the size and concentration of the filler, the refractive index of the components, the composite thickness, surface roughness, and the dispersion of the filler particles in the polymer matrix. Our nanocomposites containing unmodified NC, and NC bearing poly(methacrylic acid) grafts showed a high transparency, only 10% lower than that of the neat epoxy system. Similar results were obtained by Lee et al. [59] for millimeter thick cellulose nanofiber/epoxy laminates and by Fan et al. [60] for a biobased epoxy resin containing SiO_2_-hybridized nanocellulose.

**Figure 7:**
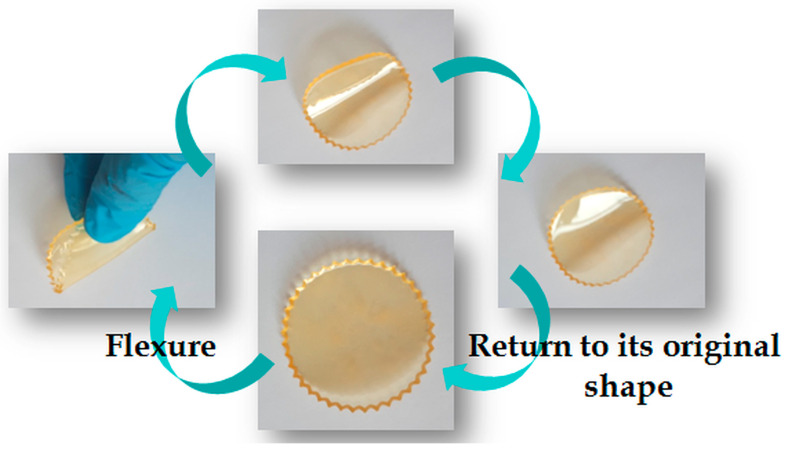
Flexibility of the obtained epoxy/NCs nanocomposites demonstrated by their returning to the initial form.

The strong increase in the storage modulus of the E/NC/SM nanocomposite as compared to the neat epoxy matrix could indicate a poorer flexibility or even brittleness [61,62]. However, the E/NC/SM nanocomposite film showed good flexibility when subjecting to repeated manual flexures, returning to its initial form in about 70 s (Figure 7).

As one can observe from Table 2, the presence of ungrafted and silane-grafted NCs determined a slight increase in the *T_g_* values as compared with the neat epoxy matrix, which correlated well with the increase in the calculated crosslink density [48]. Thus, a higher crosslink density of the nanocomposites generates a decrease in the free volume within the neat epoxy system, restricting the free movement of the polymer chain segments in these nanocomposites, which leads to an increase of in the *T_g_* values [51].

The magnitude of tan δ peak was considerably reduced, by up to 42%, for the nanocomposites with ungrafted and grafted NCs as compared to the neat epoxy matrix (Table 2). This is consistent with a less viscous behaviour and improved thermomechanical stability for the obtained nanocomposites due to the addition of NCs.

### 2.5. SEM Analysis of Neat Epoxy System and Epoxy/NC Nanocomposites

**Figure 8:**
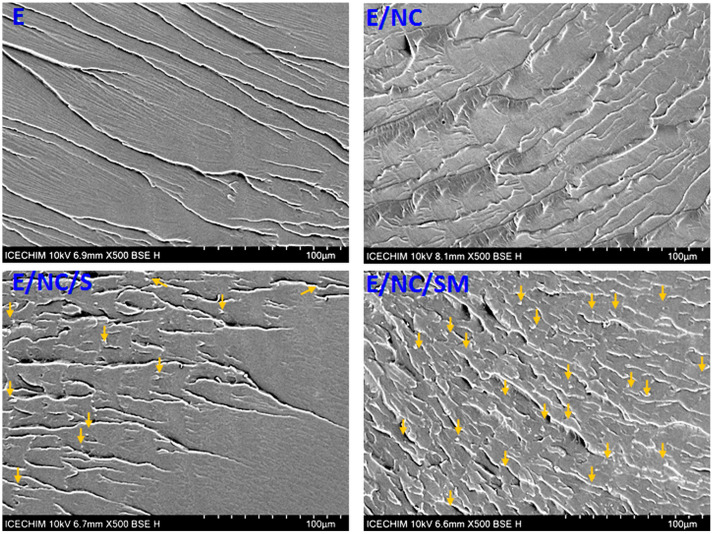
SEM micrographs of cryo-fractured surfaces of cured neat epoxy matrix and epoxy/NCs nanocomposites (×500).

The SEM images of cryo-fractured samples from the cured neat epoxy matrix and its nanocomposites with NCs are shown in Figure 8.

The microstructure of the cured neat epoxy matrix shows a brittle behavior in liquid nitrogen with a completely smooth and flat surface and evidence of branched river-like fracture propagation (Figure 8) [38]. Contrarily, the epoxy nanocomposites showed remarkably different fracture microstructures (Figure 8). Thus, the fracture surface of all nanocomposites was rough, with a leaf-like uniform pattern consisting of both crack-deflections and stress-whitening areas, with the latter being responsible for the restriction of fracture growth through the epoxy matrix (Figure 8) [38]. No pulled out NCs or voids were detected on the nanocomposite surface micrographs, revealing a good interfacial adhesion with the epoxy matrix.

Nevertheless, a distinct morphology was noticed in the case of E/NC/SM nanocomposite, probably due to the presence of poly(metacrylic acid) on the NCs surface which led to an increased interface volume and compatibility with the epoxy matrix [63]. Additionally, this sample showed a large number of stress-whitening zones due to the plastic deformation of the epoxy matrix around the SM grafted NCs [38]. Indeed, the nanocomposite containing NC/SM showed the best thermal and thermo-mechanical properties, thus confirming this observation. Moreover, few individual fractured NC ends (indicated by the yellow arrows) could be localized mainly on the surface of the E/NC/S and E/NC/SM nanocomposites, indicating that most of the NCs were embedded within the cross-linked network of the epoxy matrix and almost fully covered by the epoxy polymer chains (Figure 8).

**Figure 9:**
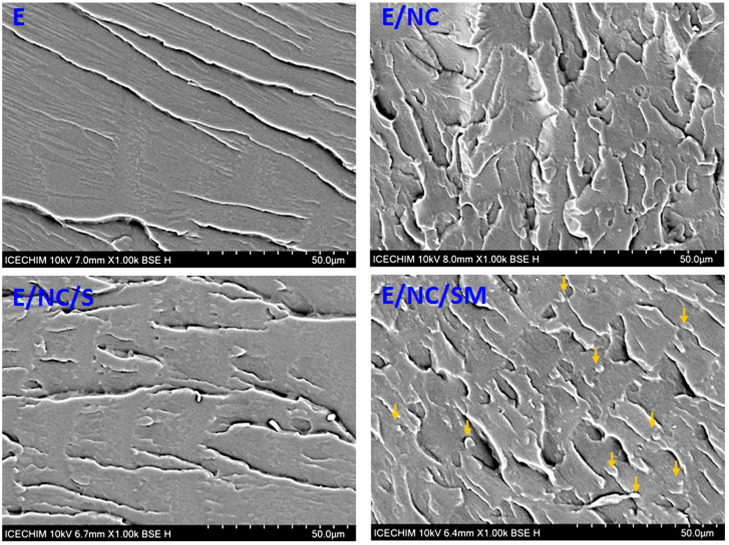
SEM micrographs of cryo-fractured surfaces of cured neat epoxy matrix and epoxy/NCs nanocomposites (×1000).

The higher magnification SEM images of the fractured samples confirm the previous observations. More than that, a homogenous distribution of the grafted NCs was noticed in the epoxy matrix due to the good miscibility between the surface-treated nanofibrillated cellulose and the epoxy matrix (Figure 9).

### 2.6. BDS Analysis of Neat Epoxy System and Epoxy/NC Nanocomposites

Generally, epoxy resins-based materials have widespread use in many applications because of their favorable mechanical properties, excellent chemical resistance, and especially good electrical insulation. Considering this last characteristic, the obtained bio-based nanocomposites were investigated through BDS in order to assess their suitability as potential candidates for replacing the petroleum-based thermosets in electronic applications.

**Figure 10:**
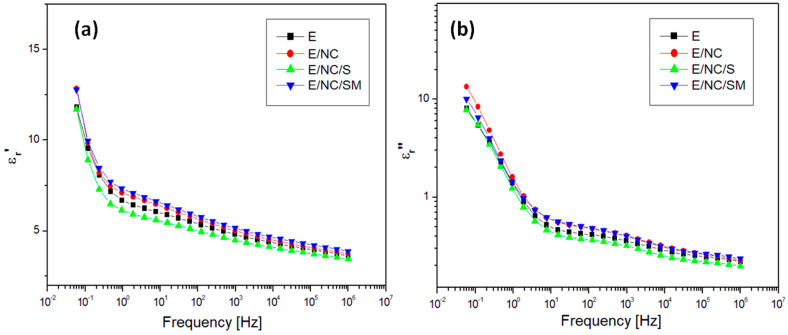
Dielectric spectra of bio-based epoxy and its nanocomposites: real part of the complex relative permittivity(ε_r_′) (**a**) and imaginary part of the complex relative permittivity (ε_r_″) (**b**).

The dielectric spectra of the neat bio-based epoxy system and epoxy nanocomposites containing differently treated NCs are shown in Figure 10a,b. The ε_r_′ decreased with frequency for all the samples in the studied range. The ε_r_′ values are mainly determined by the dielectric response of bio-based epoxy. The presence of NC, the epoxy–NC interface, and the silane treatment seem to have a small influence on the dielectric activity of the bio-epoxy material as previously observed for common epoxy nanocomposites [64]. The decrease was steeper for frequencies less than 1 Hz, mainly due to the electrode polarization, but a very limited influence of direct-current (dc) conductivity can also be possible. Although the difference between the values of the real permittivity of the nanocomposites is small, a lower ε_r_′ value was observed for the E/NC/S compared with the E reference and the other nanocomposites, regardless of the frequency. The lower permittivity of this nanocomposite as compared to the unfilled resin is unexpected and may be explained by the silane treatment of NC which prevents the absorption of water and reduces the number of hydrophilic OH on the surface of nanocellulose. A similar behavior was not observed in the case of E/NC/SM due to the polymethacrylic acid lateral chains and their hydrophilic carboxyl groups. It is worth remarking that the ε_r_’s values obtained in the case of bio-based epoxy and its nanocomposites are very close to those reported for both common and bio-based epoxy resins in a similar frequency range [65,66,67].

The dielectric loss (ε_r_″) values are higher for all the samples at low frequencies due to electrode polarization (Figure 10b). A relaxation peak was observed for all the samples at about 10^2^ Hz. This peak corresponds to the α-relaxation at the glass-to-rubber transition [68,69]. Interfacial polarization due to the accumulation of charges at the polymer–NC interface has a small influence on the variation of ε_r_″ with frequency, probably because it is hidden by the low space charge level. Similar to the ε_r_′, slightly smaller ε_r_″ values were obtained for the E/NC/S nanocomposite as compared to the E reference and the other nanocomposites, showing slightly lower losses regardless of the frequency. A very small shoulder may be seen at high frequencies (10^5^–10^6^ Hz), and it may be attributed to the β-relaxation of the dipoles associated with the polar groups of the bio-epoxy resin [68].

The small influence of the NCs, regardless of the surface treatment, on the dielectric properties of the bio-based epoxy matrix correlated with the improved thermal and mechanical properties. This shows that these nanocomposites may be recommended as suitable materials for flexible electronic devices.

## 3. Materials and Methods

### 3.1. Materials

ELO was kindly supplied by TRAQUISA (Barcelona, Spain), while IA (≥99.9%), and THF (≥99.9%, inhibitor-free) were acquired from Sigma-Aldrich (Merck KGaA, Darmstadt, Germany) and used as received. Microcrystalline cellulose (MCC, 20 μ) from Sigma-Aldrich (Merck KGaA, Darmstadt, Germany) was used as raw material for NC’s isolation.

### 3.2. Isolation and Surface Treatment of NC

According to our previous report, NC was obtained from microcrystalline cellulose (MCC) via a high-pressure mechanical treatment [70]. Briefly, a 2 wt% MCC suspension in distilled water was passed 12 times at a pressure of 200 MPa through an LM20 type microfluidizer (Microfluidics, Westwood, MA, USA) with the formation of a cellulose gel. Further, the obtained cellulose gel was frozen, then freeze-dried, and finally milled using an ultra-centrifugal mill (ZM 200, Retsch GmbH & Co., Düsseldorf, Germany) at a speed of 6000 min^−1^, when a nanofibrillated cellulose (NC) powder was obtained. The resulting NC was firstly treated with a 5 wt% γ-methacryloyloxypropyltrimethoxysilane (S) water solution at 50 °C, under strong magnetic stirring, for 4 h for generating active sites on its surface and then subjected to graft polymerization with methacrylic acid (M). The graft polymerization reaction was carried out using acetone as solvent and AIBN as initiator by mixing all components together and allowing them to react under reflux for 4 h. The silane modified (NC/S) and methacrylic acid grafted (NC/SM) nanocellulose were further washed with distilled water, freeze-dried, and milled (Figure 1a). The results from the structural analysis of NC/S and NC/SM allowed us to conclude that both the silane and graft polymerization treatments were highly efficient [70].

### 3.3. Preparation of Epoxy System and Epoxy Nanocomposites

All formulations were prepared by maintaining a stoichiometric ratio of 1:1 between the epoxy groups of ELO and the acid groups of IA, while the weight ratio between THF and IA was set at 3:1. The content of NC, NC/S, and NC/SM, respectively, was maintained constant at 0.75 wt% in all the epoxy nanocomposites. For the preparation of the ELO-based nanocomposites, the IA was first solubilized in THF under magnetic stirring at 50 °C until a transparent solution was obtained. Simultaneously, the desired amount of unmodified or surface-modified NCs was dispersed in the ELO resin and homogenized at 80 °C for 30 min under vigorous stirring. Afterwards, the THF/IA solution was poured over the ELO/NC mixture, and the stirring continued for another 30 min. Subsequently, the resulting nanocomposites were poured into silicon molds, degassed under vacuum at ambient temperature, and cured in an oven at 90 °C for 90 min, 120 °C for 60 min, and 140 °C for another 60 min. A neat epoxy system, without NCs, was prepared following the same protocol and served as a control. The schematic illustration of the nanocomposites’ preparation protocol is displayed in Figure 1b while the samples’ code names, together with the curing conditions, are summarized in Table 3. It is important to state that both the neat epoxy and the nanocomposites were transparent before and after curing, indicating a good macroscopic homogeneity (Figure 6a).

### 3.4. Characterization of Neat Epoxy System and Epoxy/NC Nanocomposites

#### 3.4.1. Fourier-Transformed Infrared (FT-IR) Spectroscopy

The FT-IR absorbance spectra of pristine IA and ELO, the neat epoxy system and epoxy nanocomposites obtained following the curing protocol were recorded using a Jasco FTIR 6300 instrument (JASCO Int. Co., Ltd., Tokyo, Japan) in attenuated total reflectance (ATR) mode over a wavenumber range from 4000 to 400 cm^−1^. The resolution and number of scanning cycles were 4 cm^−1^ and 32, respectively.

#### 3.4.2. Temperature-Modulated Differential Scanning Calorimetry (TMDSC)

TMDSC analysis was employed for monitoring the efficiency of the curing protocol used in the preparation of the neat epoxy system and epoxy nanocomposites and was conducted by using a TA Instruments DSC Q2000 equipment (TA Instruments Inc., New Castle, DE, USA) under helium flow (25 mL/min), with the underlying rate of 10 °C/min, amplitude of 0.8 °C/min, and a period of 30 s as follows: equilibrating at −85 °C; isothermal for 5 min; heating from −85 °C to 305 °C.

#### 3.4.3. Thermogravimetric Analysis (TGA)

The thermal stability of the cured neat epoxy matrix and epoxy nanocomposites was evaluated using TA Q5000 equipment (TA Instruments Inc., New Castle, DE, USA). The samples were analyzed in nitrogen at a flow rate of 50 mL min^−1^ during heating from room temperature to 750 °C at 10 °C min^−1^. All samples were run in duplicate to verify the reproducibility, and the average values, along with the standard deviation, were reported.

#### 3.4.4. Dynamic Mechanical Analysis (DMA)

The viscoelastic behavior of cured epoxy nanocomposites was assessed using a TA Instruments DMA Q800 (TA Instruments Inc., New Castle, DE, USA). The tests were performed in multi-frequency strain mode (tension clamp) by heating the bar samples with dimensions of 13 × 7 × 1.2 mm^3^ (length × width × thickness) from −30 to 90 °C at a heating rate of 3 °C min^−1^ and an oscillation frequency of 1.0 Hz.

The crosslink density (ν) was determined in accordance with the Flory theory of rubber elasticity [71], using Equation (1):

ν = E′/3RT(1)

where E′ is the storage modulus of the neat epoxy system and epoxy nanocomposites, respectively, in the rubbery region at T = (T_g_ + 50) in Kelvin, and R is the ideal gas constant.

#### 3.4.5. Transmittance Measurements

The transmittance spectra of cured bio-based epoxy matrix and epoxy/NCs nanocomposites were recorded in the visible region (300–800 nm) on a JASCO V570 UV-VIS-NIR spectrophotometer (Jasco Int. Co., Ltd., Tokyo, Japan), equipped with a JASCO ILN-472 (150 mm) integrating sphere.

#### 3.4.6. Scanning Electron Microscopy (SEM)

The cured epoxy nanocomposites were fractured in liquid nitrogen and then sputter-coated (Q150R Plus, Quorum, SXE, Lewes, UK) with a 5 nm layer of gold. The surface of fractured blends was visualized with a Hitachi TM4000Plus II Benchtop microscope working in a low vacuum at 10 kV accelerating voltage and a working distance of about 7 mm. The SEM images were taken using backscattered electron detector (BSE).

#### 3.4.7. Broadband Dielectric Spectroscopy (BDS)

BDS measurements were carried out with a Novocontrol Alpha-A Analyzer equipped with an Active Sample Cell ZGS over a frequency range from 5 × 10^−2^ to 1 × 10^6^ Hz. The real (εr′) and imaginary (εr″) parts of the complex relative permittivity were determined for each sample at the temperature of 30 °C. The samples were maintained for 30 min at this temperature before measurements.

## 4. Conclusions

Bio-based epoxy nanocomposites consisting of ELO, surface-modified NCs as fillers, and IA as cross-linking agent were developed for the first time. The overall results showed that NCs can be used for tailoring the mechanical properties of ELO-based systems. DSC results revealed that the addition of NCs into the bio-based epoxy network did not alter the curing mechanism and had a slight impact on the maximum peak temperature. Even at low content, NCs exhibited a favorable effect in terms of both thermal stability and mechanical behavior. As compared with the neat epoxy, the onset degradation temperature of the epoxy-based nanocomposites increased by more than 30 °C, regardless of their treatment. The nanocomposite containing NC/SM showed the best mechanical performance, exhibiting an increase in the storage modulus by more than 60% at room temperature. This indicates a better dispersion of the nanofibers and a higher adhesion between the epoxy system and the poly(methacrylic acid) grafts, which resulted in a more homogenous structure, as supported by the SEM results. A small influence of NCs on the dielectric activity of the bio-epoxy nanocomposites was observed from the dielectric measurements, thus showing the suitability of the obtained bio-based epoxy/NCs for replacing the petroleum-based thermosets in electronic applications. Therefore, the developed bio-based nanocomposites may provide a new perspective on using them as coatings or flexible films for different engineering applications.

## Figures and Tables

**Table 1:** Thermal properties of the ELO epoxy matrix and its nanocomposites with NCs.

Sample	T_d,5%_, °C	T_d,max_, °C	WL_200°C_ %	R_750°_, %
E	240.3 ± 4.95	387.7 ± 0.49	2.78 ± 0.6	0.04
E/NC	271.8 ± 2.33	388.8 ± 2.05	1.98 ± 0.13	0.33
E/NC/S	272.9 ± 2.26	388.8 ± 0.64	1.83 ± 0.12	1.52
E/NC/SM	284.2 ± 3.82	389.4 ± 1.91	1.64 ± 0.24	0.86

**Table 2:** Dynamic mechanical data for epoxy matrix and nanocomposites containing NCs.

Samples	*E*’_−25°C_ (MPa)	*E*’_0°C_ (MPa)	*E*’_25°C_ (MPa)	*E*’_50°C_ (MPa)	*T_g,E’_* (°C)	Crosslink Density (mmol cm^−3^)	tan *δ_max_*
E	745.9	814.1	315.2	16.0	22.9	1.02	0.74
E/NC	729.7	770.3	455.1	53.2	28.1	1.43	0.47
E/NC/S	801.3	846.1	409.5	46.4	24.4	1.30	0.43
E/NC/SM	977.6	1087	506.2	31.7	19.9	1.41	0.47

**Table 3:** ELO-derived nanocomposites formulations and curing conditions employed in their preparation.

Sample Code	ELO	NC	NC/S	NC/SM	Curing Conditions
E	yes	-	-	-	90 °C, 90 min; 120 °C, 60 min; 140 °C, 60 min
E/NC	yes	yes		-	
E/NC/S	yes		yes	-	
E/NC/SM	yes	-	-	yes	

## Data Availability

The data presented in this study are available on request from the corresponding authors.

## References

[1] Baroncini E.A., Yadav S.K., Palmese G.R., Stanzione J.F. (2016). Recent advances in bio-based epoxy resins and bio-based epoxy curing agents. J. Appl. Polym. Sci..

[2] Ma S., Li T., Liu X., Zhu J. (2015). Research progress on bio-based thermosetting resins. Polym. Int..

[3] Ocando C., Ecochard Y., Decostanzi M., Caillol S., Avérous L. (2020). Dynamic network based on eugenol-derived epoxy as promising sustainable thermoset materials. Eur. Polym. J..

[4] Liu J., Wang S., Peng Y., Zhu J., Zhao W., Liu X. (2021). Advances in sustainable thermosetting resins: From renewable feedstock to high performance and recyclability. Prog. Polym. Sci..

[5] Rad E.R., Vahabi H., de Anda A.R., Saeb M.R., Thomas S. (2019). Bio-epoxy resins with inherent flame retardancy. Prog. Org. Coat..

[6] Ma Y., Liu H., Wu J., Yuan L., Wang Y., Du X., Wang R., Marwa P.W., Petlulu P., Che X. (2019). The adverse health effects of bisphenol A and related toxicity mechanisms. Environ. Res..

[7] Waidyanatha S., Gaudette N.F., Hong Y., Fennell T.R. (2014). Formation of Epichlorohydrin, a Known Rodent Carcinogen, Following Oral Administration of 1,3-Dichloro-2-propanol in Rats. Chem. Res. Toxicol..

[8] Nabipour H., Wang X., Song L., Hu Y. (2023). Synthesis of a bio-based and intrinsically anti-flammable epoxy thermoset and the application of its carbonized foam as an efficient CO_2_ capture adsorbent. Mater. Today Sustain..

[9] Muhamad M.S., Salim M.R., Lau W.J., Yusop Z. (2016). A review on bisphenol A occurrences, health effects and treatment process via membrane technology for drinking water. Environ. Sci. Pollut. Res..

[10] De B., Gupta K., Mandal M., Karak N. (2014). Biodegradable Hyperbranched Epoxy from Castor Oil-Based Hyperbranched Polyester Polyol. ACS Sustain. Chem. Eng..

[11] Kumar S., Samal S.K., Mohanty S., Nayak S.N. (2019). Curing kinetics of bio-based epoxy resin-toughened DGEBA epoxy resin blend. J. Therm. Anal. Calorim..

[12] Wan J., Zhao J., Zhang X., Fan H., Zhang J., Hu D., Jin P., Wang D.Y. (2020). Epoxy thermosets and materials derived from bio-based monomeric phenols: Transformations and performances. Prog. Polym. Sci..

[13] Saikia A., Karak N. (2017). Renewable resource based thermostable tough hyperbranched epoxy thermosets as sustainable materials. Polym. Degrad. Stab..

[14] Schwaiger M., Resch-Fauster K. (2022). Mechanical flexible epoxy resins with 100% bio-based carbon content based on epoxidized vegetable oils. J. Appl. Polym. Sci..

[15] Chen C.H., Tung S.H., Jeng R.J., Abu-Omar M.M., Lin C.H. (2019). A facile strategy to achieve fully bio-based epoxy thermosets from eugenol. Green Chem..

[16] Reinhardt N., Breitsameter J.M., Drechsler K., Rieger B. (2022). Fully Bio-Based Epoxy Thermoset Based on Epoxidized Linseed Oil and Tannic Acid. Macromol. Mater. Eng..

[17] Xu C., Zheng Z., Wu W., Fu L., Lin B. (2019). Design of healable epoxy composite based on β-hydroxyl esters crosslinked networks by using carboxylated cellulose nanocrystals as crosslinker. Compos. Sci. Technol..

[18] Li Y., Xiao F., Moon K.-S., Wong C.P. (2005). Novel curing agent for lead-free electronics: Amino acid. J. Polym. Sci. Part A Polym. Chem..

[19] Sahoo S., Khandelwal V., Manik G. (2018). Development of completely bio-based epoxy networks derived from epoxidized linseed and castor oil cured with citric acid. Polym. Adv. Technol..

[20] Di Mauro C., Genua A., Mija A. (2022). Fully bio-based reprocessable thermosetting resins based on epoxidized vegetable oils cured with itaconic acid. Ind. Crops. Prod..

[21] Lee S.H., MdTahir P., Lum W.C., Tan L.P., Bawon P., Park B.-D., Osman Al Edrus S.S., Abdullah U.H. (2020). A Review on Citric Acid as Green Modifying Agent and Binder for Wood. Polymers.

[22] Ma S., Liu X., Jiang Y., Tang Z., Zhang C., Jin Z. (2013). Bio-based epoxy resin from itaconic acid and its thermosets cured with anhydride and comonomers. Green. Chem..

[23] Naderi P., Kabiri K., Jahanmardi R., Zohuriaan-Mehr M.J. (2020). Preparation of itaconic acid bio-based cross-linkers for hydrogels. J. Macromol. Sci. A..

[24] Tarabanko N., Baryshnikov S.V., Kazachenko A.S., Miroshnikova A.V., Skripnikov A.M., Lavrenov A.V., Taran O.P., Kuznetsov B.N. (2022). Hydrothermal hydrolysis of microcrystalline cellulose from birch wood catalyzed by Al_2_O_3_-B_2_O_3_ mixed oxides. Wood Sci. Technol..

[25] Ahmad H., Anguilano L., Fan M. (2022). Microstructural architecture and mechanical properties of empowered cellulose-based aerogel composites via TEMPO-free oxidation. Carbohydr. Polym..

[26] Abdelhamid H.N., Mathew A.P. (2022). Cellulose-Based Nanomaterials Advance Biomedicine: A Review. Int. J. Mol. Sci..

[27] Wang B., Zhang W., Sun J., Lai C., Ge S., Guo H., Liu Y., Zhang D. (2023). A micro/nano-multiscale hierarchical structure strategy to fabricate highly conducting films for electromagnetic interference shielding and energy storage. J. Mater. Chem. A.

[28] Vatansever E., Arslan D., Nofar M. (2019). Polylactide cellulose-based nanocomposites. Int. J. Biol. Macromol..

[29] Frone A.N., Panaitescu D.M., Chiulan I., Gabor A.R., Nicolae C.A., Oprea M. (2019). Thermal and mechanical behavior of biodegradable polyester films containing cellulose nanofibers. J. Therm. Anal. Calorim..

[30] Zhang W., Zhang G., Lu X.-a., Wang J., Wu D. (2021). Cellulosic nanofibers filled poly(β-hydroxybutyrate): Relations between viscoelasticity of composites and aspect ratios of nanofibers. Carbohydr. Polym..

[31] Panaitescu D.M., Lupescu I., Frone A.N., Chiulan I., Nicolae C.A., Tofan V., Stefaniu A., Trusca R. (2017). Medium chain-length polyhydroxyalkanoate copolymer modified by bacterial cellulose for medical devices. Biomacromolecules.

[32] Platnieks O., Sereda A., Gaidukovs S., Thakur V.K., Barkane A., Gaidukova G.M., Filipova I., Ogurcovs A., Fridrihsone V. (2021). Adding value to poly (*butylene succinate*) and nanofibrillated cellulose-based sustainable nanocomposites by applying masterbatch process. Ind. Crops Prod..

[33] Bangar S.P., Whiteside W.S. (2021). Nano-cellulose reinforced starch bio composite films—A review on green composites. Int. J. Biol. Macromol..

[34] Frone A.N., Nicolae C.A., Gabor R.A., Panaitescu D.M. (2015). Thermal properties of water-resistant starch—Polyvinyl alcohol films modified with cellulose nanofibers. Polym. Degrad. Stab..

[35] Khelifa F., Habibi Y., Bonnaud L., Dubois P. (2016). Epoxy monomers cured by high cellulosic nanocrystals loadings. ACS Appl. Mater. Interfaces.

[36] Peng S.X., Shrestha S., Yoo Y., Youngblood J.P. (2017). Enhanced dispersion and properties of a two-component epoxy nanocomposite using surface modified cellulose nanocrystals. Polymer.

[37] Zhao J., Li Q., Zhang X., Xiao M., Zhang W., Lu C. (2017). Grafting of polyethylenimine onto cellulose nanofibers for interfacial enhancement in their epoxy nanocomposites. Carbohydr. Polym..

[38] Laghaei R., Hejazi S.M., Fashandi H., Akbarzadeh S., Shaghaghi S., Shamaei-Kashani A., Jahanara B., Shahsavari E. (2023). Reinforcement contribution of cellulose nanocrystals (CNCs) to tensile properties and fracture behavior of triaxial E-glass fabric/epoxy composites. Compos. Part A Appl. Sci. Manuf..

[39] Trinh B.M., Mekonnen T. (2018). Hydrophobic esterification of cellulose nanocrystals for epoxy reinforcement. Polymer.

[40] Yeo J., Kim O.Y., Hwang S. (2017). The effect of chemical surface treatment on the fracture toughness of microfibrillated cellulose reinforced epoxy composites. J. Ind. Eng. Chem..

[41] Emami Z., Meng Q., Pircheraghi G., Manas-Zloczower I. (2015). Use of surfactants in cellulose nanowhisker/epoxy nanocomposites: Effect on filler dispersion and system properties. Cellulose.

[42] Shibata M., Teramoto N., Makino K. (2011). Preparation and Properties of Biocomposites Composed of Epoxidized Soybean Oil, Tannic Acid, and Microfibrillated Cellulose. J. Appl. Polym. Sci..

[43] Shibata M., Nakai K. (2010). Preparation and Properties of Biocomposites Composed of Bio-Based Epoxy Resin, Tannic Acid, and Microfibrillated Cellulose. J. Polym. Sci. B Polym. Phys..

[44] Wu G., Liu D., Liu G., Chen J., Huo S., Kong Z. (2015). Thermoset nanocomposites from waterborne bio-based epoxy resin and cellulose nanowhiskers. Carbohydr. Polym..

[45] Kwon Y.R., Kim H.C., Kim J.S., Chang Y.W., Park H., Kim D.H. (2022). Itaconic-acid-based superabsorbent polymer with high gel strength and biocompatibility. Polym. Int..

[46] Ge H., Hua T., Wang J. (2017). Preparation and characterization of poly (itaconic acid)-grafted crosslinked chitosan nanoadsorbent for high uptake of Hg^2+^ and Pb^2+^. Int. J. Biol. Macromol..

[47] Necolau M.I., Damian C.M., Olaret E., Iovu H., Balanuca B. (2022). Comparative Thermo-Mechanical Properties of Sustainable Epoxy Polymer Networks Derived from Linseed Oil. Polymers.

[48] Ding C., Peter S., Shuttleworth P.S., Makin S., Clark J.H., Avtar S., Matharu A.S. (2015). New insights into the curing of epoxidized linseed oil with dicarboxylic acids. Green Chem..

[49] Todorovic A., Resch-Fauster K., Mahendran A.R., Oreski G., Kern W. (2021). Curing of epoxidized linseed oil: Investigation of the curing reaction with different hardener types. J. Appl. Polym. Sci..

[50] Hameed N., Bavishi J., Parameswaranpillai J., Salim N.V., Joseph J., Madrasd G., Foxab B.L. (2015). Thermally flexible epoxy/cellulose blends mediated by an ionic liquid. RSC Adv..

[51] Thompson L., Nikzad M., Sbarski I., Yu A. (2022). Esterified cellulose nanocrystals for reinforced epoxy nanocomposites. Prog. Nat. Sci..

[52] Roszowska-Jarosz M., Masiewicz J., Kostrzewa M., Kucharczyk W., Zurowski W., Kucińska-Lipka J., Przybyłek P. (2021). Mechanical Properties of Bio-Composites Based on Epoxy Resin and Nanocellulose Fibres. Materials.

[53] Yu Q., Liang Y., Cheng J., Chen S., Zhang A., Miao M., Zhang D. (2017). Synthesis of a Degradable High-Performance Epoxy-Ended Hyperbranched Polyester. ACS Omega.

[54] Saba N., Safwan A., Sanyang M.L., Mohammad F., Pervaiz M., Jawaid M., Alothman O.Y., Sain M. (2017). Thermal and dynamic mechanical properties of cellulose nanofibers reinforced epoxy composites. Int. J. Biol. Macromol..

[55] Nair S.S., Kuo P.Y., Chen H., Yan N. (2017). Investigating the effect of lignin on the mechanical, thermal, and barrier properties of cellulose nanofibril reinforced epoxy composite. Ind. Crops Prod..

[56] Pandurangan M.T., Kanny K. (2020). Study of Curing Characteristics of Cellulose Nanofiber-Filled Epoxy Nanocomposites. Catalysts.

[57] Neves Monteiro S., Salgado de Assis F., Ferreira C.L., Tonini Simonassi N., Pondé Weber R., Souza Oliveira M., Colorado H.A., Camposo Pereira A. (2018). Fique Fabric: A Promising Reinforcement for Polymer Composites. Polymers.

[58] Nair S.S., Dartiailh C., Levin D.B., Yan N. (2019). Highly Toughened and Transparent Biobased Epoxy Composites Reinforced with Cellulose Nanofibrils. Polymers.

[59] Lee K., Kwon G., Jeon Y., Jeon S., Hong C., Choung J.W., You J. (2022). Toward millimeter thick cellulose nanofiber/epoxy laminates with good transparency and high flexural strength. Carbohydr. Polym..

[60] Fan X., Miao J.-T., Yuan L., Guan Q., Gu A., Liang G. (2018). Preparation and origin of thermally resistant biobased epoxy resin with low internal stress and good UV resistance based on SiO_2_ hybridized cellulose for light emitting diode encapsulation. Appl. Surf. Sci..

[61] Voo R., Mariatti M., Sim L. (2012). Flexibility improvement of epoxy nanocomposites thin films using various flexibilizing additives. Compos. B Eng..

[62] Unnikrishnan K.P., Thachil E.T. (2006). Toughening of epoxy resins. Des. Monomers Polym..

[63] Necolau M., Balanuca B., Frone A.N., Damian C.M. (2022). Tailoring an effective interface between nanocellulose and epoxidized linseed oil network through functionalization. ACS Omega.

[64] Singha S., Thomas M.J. (2008). Dielectric properties of epoxy nanocomposites. IEEE Trans. Dielectr. Electr. Insul..

[65] Wang Y., Zhu L., Zhou J., Jia B., Jiang Y., Wang J. (2019). Dielectric properties and thermal conductivity of epoxy resin composite modified by Zn/ZnO/Al_2_O_3_ core–shell particles. Polym. Bull..

[66] Qiang D., Wang Y., Chen G., Andritsch T. (2018). Dielectric properties of epoxy silica and boron nitride nanocomposites and moisture/temperature influences. IET Nanodielectr..

[67] Zafar R., Gupta N. (2020). Dielectric spectroscopy of epoxy-based barium titanate nanocomposites: Effect of temperature and humidity. IET Nanodielectrics.

[68] Min D., Cui H., Hai Y., Li P., Xing Z., Zhang C., Li S. (2020). Interfacial regions and network dynamics in epoxy/POSS nanocomposites unravelling through their effects on the motion of molecular chains. Compos. Sci. Technol..

[69] Sevasti G., Stavropoulos S., Sanida A., Psarras G. (2020). A comparative study on the thermomechanical and electrical properties of carbide/or graphite/epoxy-reinforced composites. J. Therm. Anal. Calorim..

[70] Popa M.S., Frone A.N., Radu I.C., Stanescu P.O., Truşcă R., Rădiţoiu V., Nicolae C.A., Gabor A.R., Panaitescu D.M. (2021). Microfibrillated Cellulose Grafted with Metacrylic Acid as a Modifier in Poly(3-hydroxybutyrate). Polymers.

[71] Flory P.J. (1953). Baker lectures 1948, George Fisher Baker non-resident lectureship in chemistry at Cornell University, The Baker lectures. Principles of Polymer Chemistry.

